# Pre‐Lithiation Strategies for Next‐Generation Practical Lithium‐Ion Batteries

**DOI:** 10.1002/advs.202005031

**Published:** 2021-03-15

**Authors:** Liming Jin, Chao Shen, Qiang Wu, Annadanesh Shellikeri, Junsheng Zheng, Cunman Zhang, Jim P. Zheng

**Affiliations:** ^1^ Clean Energy Automotive Engineering Center and School of Automotive Studies Tongji University Shanghai 201804 China; ^2^ Aero‐Propulsion, Mechatronics and Energy Center Florida State University Tallahassee FL 32310 USA; ^3^ Department of Electrical Engineering University at Buffalo The State University of New York Buffalo NY 14260 USA

**Keywords:** active lithium loss, Coulombic efficiency, lithium ion batteries, prelithiation, solid electrolyte interphase

## Abstract

Next‐generation Li‐ion batteries (LIBs) with higher energy density adopt some novel anode materials, which generally have the potential to exhibit higher capacity, superior rate performance as well as better cycling durability than conventional graphite anode, while on the other hand always suffer from larger active lithium loss (ALL) in the first several cycles. During the last two decades, various pre‐lithiation strategies are developed to mitigate the initial ALL by presetting the extra Li sources to effectively improve the first Coulombic efficiency and thus achieve higher energy density as well as better cyclability. In this progress report, the origin of the huge initial ALL of the anode and its effect on the performance of full cells are first illustrated in theory. Then, various pre‐lithiation strategies to resolve these issues are summarized, classified, and compared in detail. Moreover, the research progress of pre‐lithiation strategies for the representative electrochemical systems are carefully reviewed. Finally, the current challenges and future perspectives are particularly analyzed and outlooked. This progress report aims to bring up new insights to reassess the significance of pre‐lithiation strategies and offer a guideline for the research directions tailored for different applications based on the proposed pre‐lithiation strategies summaries and comparisons.

## Graphical abstract

This progress report reassesses the significance of pre‐lithiation strategies for the next generation lithium ion batteries and offers a guideline for the research directions tailored for different applications based on the proposed pre‐lithiation strategies summaries and comparisons.

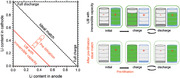

## 1 Introduction

Rechargeable Li‐ion batteries (LIBs) are one of the most widely used electrochemical energy storage systems nowadays due to their high energy density, high operating voltage, no memory effect, and minimal self‐discharge.^[^ ^1^ ^]^ Generally, the commercial LIBs are composed of graphite as anode coupled with layered transition metal oxide (e.g., LiCoO_2_, Li(CoMnNi)O_2_, etc.) or olivine type (e.g., LiFePO_4_) materials as the cathode in a Li‐containing organic electrolyte. Despite the advantages such as lower working potential (<0.1 V vs Li) and eco‐friendliness,^[^ ^1^, ^2^, ^3^ ^]^ conventional graphite anode in LIBs is difficult to completely fulfill the requirements of the emerging market of (hybrid) electric vehicles due to its limited capacity (372 mAh g^−1^). Therefore, the research activities are highly accelerated to develop alternative anodes with appreciative potential, high capacity, and large power capability as well as cycling durability to satisfy the need for the next‐generation LIBs.^[^ ^1^ ^]^ The continuous progress in the last several decades achieved by the development of novel and/or enhanced materials have led to considerable improvements in terms of higher energy density, larger power density, longer cycle life, as well as higher safety. ^[^ ^4^, ^5^, ^6^, ^7^, ^8^, ^9^, ^10^ ^]^

In the last several decades, numerous novel anode materials (e.g., low‐potential intercalation/insertion, conversion, and alloying type anode materials) were proposed as the anode candidates for next‐generation LIBs, which generally exhibited desirable potential range, higher capacity, superior rate performance as well as better cycling durability than graphite. However, these materials all suffer from huge initial active lithium loss (ALL) during cycling owing to several sources for different energy storage mechanisms as shown in:^[^ ^11^, ^12^ ^]^ (*I*) the electrolyte decomposes and form solid electrolyte interface (SEI), irreversible parasitic reactions occur (such as Li_2_O formation for some metal oxides), etc., consuming a large amount of Li irreversibly and causing huge initial ALL in the first several cycles; (*II*) unstable SEI formation, dead Li generation, and electrode volume variation upon cycling will further consume the Li in the continuously cycling. As we know, Li ions are charge carriers in LIBs shuttling back and forth between cathode and anode through the electrolyte. Therefore, Li‐ions consumption in the cycling would directly cause capacity degradation and eliminating the ALL is critical and essential before the full‐cell assembling when using such novel anode materials for next‐generation LIBs.

**Figure 1:**
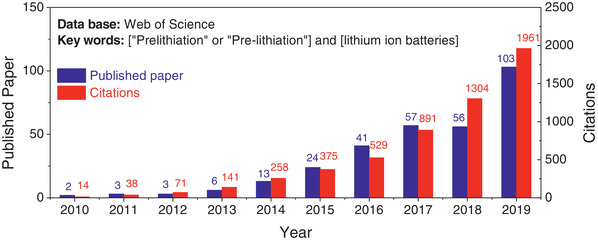
Distribution of pre‐lithiation in lithium ion batteries‐related papers by year in the web of science core collection, from 2010 to 2019. The search strategy was by topic: “Prelithiation” or “Pre‐lithiation” and lithium ion batteries.

During these years, researchers have paid increasing attention for improving cycling durability by nanostructured material designs and interface engineering, which could however exacerbate the initial ALL due to the large specific surface area (SSA) for increasing SEI formation or other side reactions in nanomaterials.^[^ ^13^ ^]^ On the other hand, many scientific literature abounds in studies focusing on the performance of active materials in half‐cell configuration to avoid the detrimental effects of initial ALL to the batteries in practical level.^[^ ^2^ ^]^ In the last decade, the novel anode materials for next‐generation LIBs came to commercialization so that the researches of pre‐lithiation technologies, one of the most promising strategies to mitigate the huge initial ALL, exhibit an exponential growth from 2010 as shown in **Figure** **1**. There are several latest reviews summarizing the progress of pre‐lithiation/pre‐sodiation strategies on different energy storage systems, which provides the broad knowledge about pre‐lithiation/pre‐sodiation.^[^ ^14^, ^15^ ^]^ In this progress report, we pay our particular attention to next generation LIBs with novel anode materials. Moreover, some theoretical analysis and strategy classification have been also discussed. We will first illustrate the origins of initial ALL for various types of anodes and the effects of the initial ALL on the capacity of the full cell, then emphasize on summaries, classifications, and comparisons of the current pre‐lithiation strategies for eliminating the initial ALL, provide the application progress of pre‐lithiation strategies in two representative systems, i.e., silicon (Si)‐based batteries, Li‐ion sulfur batteries (LISB), and finally point out the current challenges and outlook the desirable pre‐lithiation strategies for future researches and applications. This work aims to bring up new insights to reassess the significance of pre‐lithiation strategies for the next generation LIBs and offer a guideline for the research directions based on the proposed pre‐lithiation strategies.

## 2 High Initial Active Lithium Loss of Anode

### 2.1 The Origin of High Initial Active Lithium Loss of Anode

**Figure 2:**
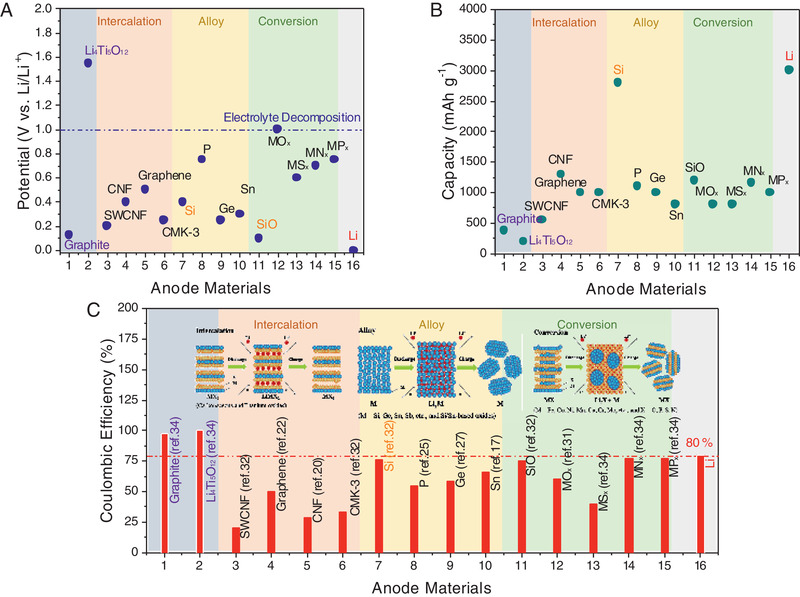
The characteristics of current representative anode materials with different energy storage mechanisms. A) The average discharge potentials of the anode materials. B) The capacities of the anode materials. C) The first Coulombic efficiencies of anode materials. Reproduced with permission.^[^ ^26^ ^]^ Copyright 2018, Steinkopff‐Verlag. Note: all the data here is generally collected from specific cited papers not the theoretical values, which exhibit some difference from different references.

The high initial ALL of anode occurs in the first several cycles, in which the de‐lithiation capacity is much lower than lithiation capacity (CE<100%), indicating some Li‐ions are left in the anode and the cyclable Li‐ions between cathode and anode are reduced. When pairing with the cathode, the reduced cyclable Li ions will inevitably result in the decreased energy density of the full cell. The inserted graph in **Figure** **2**C shows the typical anode materials with the Li storage mechanism of intercalation/insertion, conversion, and alloying. As expected, these materials all deliver relatively low potential (significantly lower than conventional Li_4_Ti_5_O_12_ (LTO) anode) as shown in Figure 2A, much higher capacity than commercial graphite and LTO (Figure 2B), but the first CE of these materials is usually lower than 80% (Figure 2C). It needs to be noted that the CEs in Figure 2 are collected from half‐cell setup, which should be different from the data from full cell due to the side reactions on cathode since the pre‐lithiation mainly targets on the low CEs resulted from the ALL.^[^ ^16^ ^]^ These low CEs (in comparison with graphite and LTO) have to be addressed to enable any commercial application of these materials. ^[^ ^17^, ^18^, ^19^, ^20^, ^21^, ^22^, ^23^, ^24^, ^25^, ^26^, ^27^, ^28^, ^29^, ^30^, ^31^, ^32^, ^33^, ^34^ ^]^

**Figure 3:**
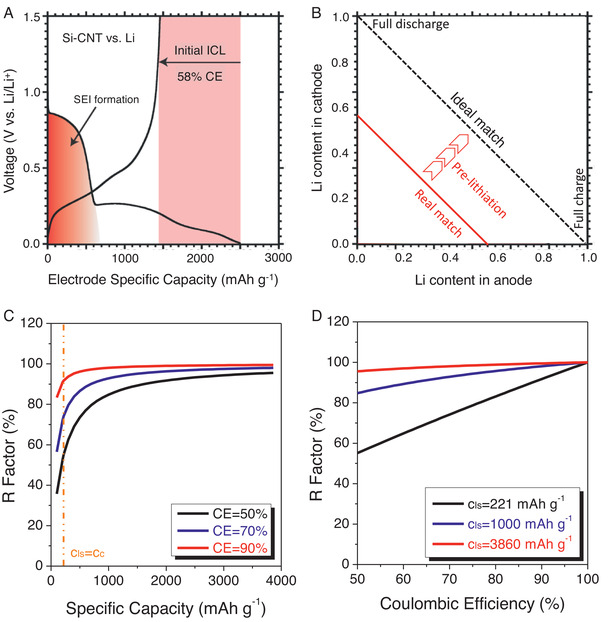
A) The representative charge and discharge profiles of novel anode materials with huge initial ALL (here is the Si‐CNT composite with the combined energy storage mechanisms of intercalation/insertion and alloying). Reproduced with permission.^[^ ^111^ ^]^ Copyright 2013, American Chemical Society. B) The effect of pre‐lithiation treatment to improve the energy density of the lithium ion batteries. C) The effects of capacities of lithium sources on the specific energy of the NMC//Si‐C system with different initial CEs. D) The R factor of NMC//Si‐C system based on the total mass of electrodes as a function of the CEs for three different specific capacities of Li sources.

Here, the mechanisms resulting in low first CE will be discussed as shown in **Figure** **3**A. In general, the causes for the initial ALL of anode could be generally classified into three categories:

#### 2.1.1 Formation of the SEI

It is well known that the electrolyte is thermodynamically unstable at lower potential versus Li^+^/Li for a negatively polarized surface. The onset potential for the electrolyte decomposition may vary and ranges often reported from ≈0.8 to 2.0 V versus Li^+^/Li. For these proposed materials, the discharge potentials are always lower than 1.0 V. Hence, electrolyte decomposition occurs irreversibly, eventually leading to the formation of a thick layer over the surface. The thickness of the SEI layer may vary from a few to tens of hundreds of nanometers. Generally, an electrolyte solution contains the mixture of cyclic (EC, PC, etc.,) and linear carbonates (DMC, DEC, etc.,) and solvated Li‐salt (LiPF_6_, LiBF_4_, LiClO_4_, etc.). Upon reduction process, there is a competition between the decomposition of the solvated ions and solvent molecules which form inorganic (e.g., Li_2_CO_3_, LiF, Li*_x_*PF*_y_*, Li*_x_*O, etc.,) and organic (e.g., (CH_2_OCO_2_Li)_2_, polyethylene oxide, polycarbonates, etc.,) by‐products over the surface of active materials and a porous organic layer is formed over the inorganic layer while using carbonaceous anodes. In another type of SEI layer (mosaic type), inorganic components were distributed within the polymetric components of SEI layer. The SEI formation will continue upon the cycling process, while the predominant Li‐consumption is noted in the first cycle only.

#### 2.1.2 Loss of Active Materials

Due to the large volume changes during cycling (especially for alloy and conversion type materials), cracking and pulverization of active particles and the surrounding matrix lead to the disconnection of some alloy particles from the conductive carbon or current collector. The breakdown of the conductive network between active particles and the carbon matrix as evidenced by the sharp rise of the internal resistance during the Li‐extraction process. Due to the large internal resistance and the isolation of the active particles, the de‐lithiation reaction was not completed, with some Li remaining in the active particles. As a result, an active lithium loss was observed. The same loss also occurs at the condition of irreversible Li metal plating, which results in dead Li. Dead Li will lose the connection with conductive network and no longer work as active materials.

#### 2.1.3 Trapping in the Host Materials

Even though Li insertion/extraction in the active materials is theoretically reversible, some Li‐ions may be permanently trapped in the alloys due to a) slow Li release kinetics, b) the formation of highly stable lithiation compounds, or c) strong bonding with less coordinated atoms at defect sites, especially for the alloy materials. A high density of defects is expected at the surface, interface, or grain boundaries in alloy particles due to the large volume change and the complicated structural transformation in the Li‐insertion/extraction process, at which Li can be trapped irreversibly. There are also some undesirable irreversible side‐reactions especially for low‐potential intercalation/insertion materials (e.g., carbon materials) and conversion materials. The carbon materials with abundant defects on the surface or at the edge and heteroatoms (e.g., N, O, F, etc.) doping would cause huge side‐reactions. A large amount of Li is stored on the defects or heteroatoms, which cannot be released again, resulting in the huge Li loss. As for the conversion materials (e.g., Co_3_O_4_), Li reacts irreversibly with many oxides to form Li_2_O at the potential of ≈0.8–1.6 V, which would cause huge Li loss and the formation of insulated Li_2_O also hinders the electrochemical activities of active materials.

All these causes are mainly occurred in the first several cycles, hence the pre‐lithiation treatment can be effective to solve these issues. Pre‐lithiation means the extra Li sources are preset into the LIB systems before cycling, which enables to mitigate the huge initial ALL of the anode. In general, pre‐lithiation treatment can be realized by cycling the anodes before matching with cathode or presenting some Li source into the system to mitigate the Li loss in the first cycle, i.e., the SEI film would be formed homogeneously on the surface of the active materials and the undesired Li loss can be compensated in advance after pre‐lithiation. Therefore, the LIB systems after pre‐lithiation treatment would exhibit high CE, and the cyclable Li‐ions are expected to shuttle back and forth between cathode and anode in a sustainable manner.

### 2.2 The Effect of the Active Lithium Loss of Anode

It is well‐known that some cyclable Li^+^ ions are consumed to form SEI on the surface of the low‐potential anode in a practical LIB, which always results in low first CE and then, in turn, leads to rapid capacity fading of the cell as shown in Figure 3B, while the reversible capacities of electrodes have no degradation.^[^ ^35^ ^]^ Therefore, when extra Li sources are added into the system, the specific capacity of the cell will increase to an ideal situation. It should be mentioned that the low CEs here particularly refer to the side reactions resulting in the ALL; while other side reactions (e.g., structure changes of the electrodes) which cannot be addressed by pre‐lithiation are not included. However, the introduction of the extra weight of Li sources will offset the specific energy gain attributed to the pre‐lithiation. In this section, to clarify the effects of the high initial ALL on the specific capacity loss of the full cell in detail, some theoretical calculation analysis is carried out, which is also useful to evaluate the efficiency of various strategies to mitigate the initial ALL.^[^ ^36^, ^37^ ^]^

On accounting of the energy storage mechanism of LIB with pre‐lithiation, cathode, anode, and Li sources for pre‐lithiation have direct effects on the specific energy theoretically, which are considered to carry out the mathematical derivation in detail. The reversible capacities of the anode, cathode and available capacity of Li source per unit separator area can be, respectively, defined as *q*_a_, *q*_c_, and *q*_ls_, which can be expressed as

(1) $$q_{a}=c_{a}m_{a}$$

(2) $$q_{c}=c_{c}m_{c}$$

(3) $$q_{\mathrm{ls}}=c_{\mathrm{ls}}m_{\mathrm{ls}}$$

where *c*_a_, *c*_c_, *c*_ls_ are specific capacities of anode, cathode, and Li source, respectively; *m*_a_, *m*_c_, and *m*_ls_ are masses per unit area of anode, cathode, and Li source layer, respectively. Moreover, the total reversible capacity of the cell (*q*_0_), CE of the anode (*ε*), and consumed capacity of Li sources for the formation of SEI (*q*_SEI_) can be obtained as

(4) $$q_{0}=q_{a}=q_{c}$$

(5) $$\varepsilon \;=\;\frac{q_{a}}{q_{a}+q_{\mathrm{SEI}}}$$

(6) $$q_{\mathrm{SEI}}=q_{\mathrm{ls}}=c_{\mathrm{ls}}m_{\mathrm{ls}}$$

Combining Equations (1)–(6), the specific energy based on the total masses of anode, cathode and Li source can be obtained as

(7) $$E=\frac{q_{0}}{m_{a}+m_{c}+m_{\mathrm{ls}}}V=\frac{1}{\frac{1}{c_{a}}+\frac{1}{c_{c}}+\frac{1-\varepsilon }{\varepsilon c_{\mathrm{ls}}}}V=\frac{1}{\frac{1}{c_{a}}+\frac{1}{c_{c}}+\frac{1}{c_{\mathrm{eff}}}}V=c_{\mathrm{cell}}\frac{c_{\mathrm{eff}}}{c_{\mathrm{eff}}+c_{\mathrm{cell}}}V=Rc_{\mathrm{cell}}V$$

where *c*_eff_ = *εc*_ls_/(1‐*ε*) is defined as the effective capacity of Li source to mitigate the active lithium loss; *c*_cell_ is the maximum specific capacity of cells without irreversible capacity, 1/*c*_cell_ = 1/*c*_a_+1/*c*_c_;^[^ ^38^ ^]^ *R* is defined as a concept called reduction factor reflecting the specific energy loss due to effects of ALL of anode.

When using a typical NMC//Si‐C system, which is composed of NMC as a cathode (221 mAh g^−1^) and Si‐C composite (960 mAh g^−1^) as an anode with an average voltage of 3.7 V, as a case to calculate the *R* factor. For an ideal situation where the CE of the anode is 100%, the maximum theoretical specific energy, which is based on specific capacities of anode and cathode, and cell voltage, can be calculated at *R* = 1, and is determined to be 665 Wh kg^−1^. However, for a nonideal condition that the initial CE of an anode is less than 100%, the extra Li source is necessary to compensate for the Li loss during the initial cycle. Figure 3C shows how the different extra Li sources could influence the specific energy. Here, we considered three different initial *CEs*, namely 50%, 70%, and 90%, and varied the specific capacities of the extra Li sources from the smallest value of 10 mAh g^−1^ (Li_2_RuO_3_) to the largest value of 3860 mAh g^−1^ (Li metal). Figure 3C shows the *R* factor as a function of the specific capacity of extra Li sources (*c*_ls_) for three different anodes having initial *CEs* of 50%, 70%, and 90%, respectively. It can be seen that the *R* factor increases with increasing *c*_ls_, and the lower *CE* results in lower *R* factor. The vertical dashed line indicates when *c*_ls_ = *c*_c_. It can also be seen that when the *c*_ls_ is greater than *c*_c_, the extra Li sources are desired to be used for improved the energy density; otherwise, over‐weight cathode is promising Li source. On the other hand, more detailed evaluations of the effects of *CEs* on the *R* factor have also been demonstrated in Figure 3D. As expected, the *R* factor demonstrates the effects of pre‐lithiation on the specific energy of the cell theoretically. However, more detailed properties of Li sources and pre‐lithiation methods should be compared and evaluated for the best choices in various systems.

## 3 Summaries, Classification, and Comparisons of Current Pre‐Lithiation Strategies

**Figure 4:**
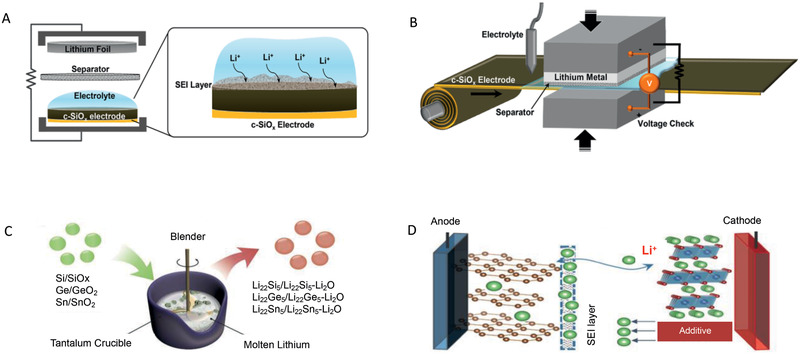
The schematic graphs of representative pre‐lithiation strategies. A) Half‐cell electrochemical method (HC‐EM). Reproduced with permission.^[^ ^39^ ^]^ Copyright 2016, American Chemical Society. B) Direct contact electrochemical method (DC‐EM). Reproduced with permission.^[^ ^39^ ^]^ Copyright 2016, American Chemical Society. C) Chemical lithiation method (CM). Reproduced with permission.^[^ ^95^ ^]^ Copyright 2017, Elsevier B.V. D) Cathode pre‐lithiation method. Reproduced with permission.^[^ ^94^ ^]^ Copyright 2016, WILEY‐VCH.

To replenish Li‐ions before cycling which would be consumed during the first cycle, the additional Li‐ions are preset into the configuration. As is known to all, LIBs have a sandwich structure with cathode, anode, and separator filled with Li containing organic electrolyte. Therefore, this section mainly summarizes various pre‐lithiation strategies and classifies these strategies based on the preset position of Li sources, i.e., cathode, anode, electrolyte, or extra components. **Figure** **4** displays typical pre‐lithiation methods, in which Figure 4A,C,D are based on the anode and cathode, respectively. All these strategies will also be compared from different points of view, in particular, e.g., required assembly condition, controllability, scalability as well as the effect of the pre‐lithiation process to the energy density of full cells.

### 3.1 Lithium Source in the Anode

**Figure 5:**
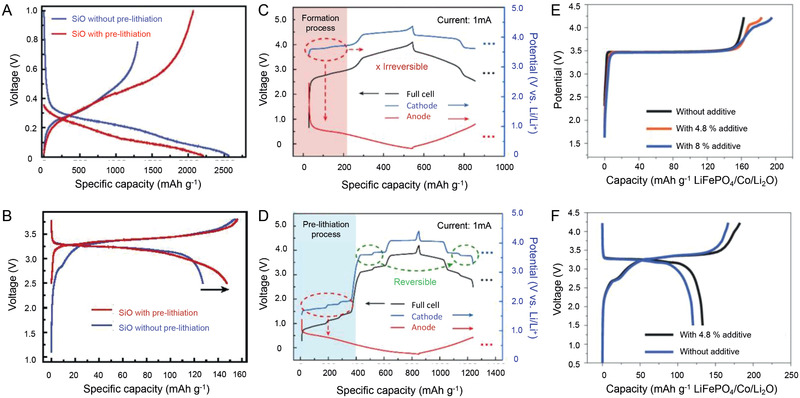
The charge and discharge profiles of A) anode and B) full cell with and without pre‐lithiation using lithium source in anode strategies. C,D) The charge and discharge profiles of cathode, anode, and full cell with and without pre‐lithiation using over‐lithiated cathode. Reproduced with permission.^[^ ^75^ ^]^ Copyright, 2019 Elsevier Ltd. The charge and discharge profiles of E) cathode and F) full cell using Li‐containing additive in cathode. Reproduced with permission.^[^ ^97^ ^]^ Copyright 2016, Nature Publishing Group.

As we discussed above, the initial ALL is caused by the irreversible electrochemical processes on the anode. Therefore, the most direct strategies to eliminate the initial ALL are the preparations of the pre‐lithiated anode by electrochemical and/or chemical strategies before pairing with the cathode. Based on the different mechanisms and methods, the strategies focusing on the anode can be divided into three categories: half‐cell electrochemical method (HC‐EM) as shown in Figure 4A, short‐circuit electrochemical method (SC‐EM) as shown in Figure 4B, and chemical method (CM) as shown in Figure 4C. After achieving the pre‐lithiation on the anode, the huge initial ALL would be addressed well, and the full cell delivers higher first cycle CE. The typical improvements for the profiles of the anode and full cell are shown in Figure 5A,B.

#### 3.1.1 HC‐EM

HC‐EM is a widely used pre‐lithiation strategy in lab studies, which can be achieved by architecting the sandwich half‐cell structure composed of working anode electrodes as cathode and Li metal foil as the anode in the presence of the electrolyte separator in between as shown in Figure 4A.^[^ ^2^, ^3^, ^4^, ^5^, ^6^, ^7^, ^8^, ^9^, ^10^, ^11^, ^12^, ^13^, ^14^, ^15^, ^16^, ^17^, ^18^, ^19^, ^20^, ^21^, ^22^, ^23^, ^24^, ^25^, ^26^, ^27^, ^28^, ^29^, ^30^, ^31^, ^32^, ^33^, ^34^, ^35^, ^36^, ^37^, ^38^, ^39^, ^40^, ^41^, ^42^, ^43^, ^44^, ^45^, ^46^, ^47^, ^48^, ^49^ ^]^ As soon as the external short circuit is constructed, the spontaneous pre‐lithiation will be initiated by the potential difference between two electrodes. More accurately, a resistor or constant current power supplier is used in the external circuit to control the speed of the pre‐lithiation and pre‐lithiation level can be determined by monitoring the final voltage or the pre‐lithiation time. After lithiation is finished, the lithiated anode can be deassembled from half‐cell and reassembled as the anode to construct full cell with the cathode.

The most important advantages of this strategy are the simplicity in lab scale, which is controllable through current as well as the end of voltage after pre‐lithiation.^[^ ^1^ ^]^ Moreover, the structure for pre‐lithiation is the same as the actual battery operation, in which the lithiation of the anode is mediated by Li‐ion flux, not direct contact with Li metal. Thus, an identical SEI layer to that generated from electrochemical cycling can be designed and formed so that the existing electrolyte technology involving additive optimization can still be utilized. However, the complex deassembling and re‐assembling operations as well as large usage of electrolytes for pre‐lithiation processes cause a huge cost to disable this strategy for practical application. More seriously, all the processes must be carried out in a moisture‐free atmosphere, while trace amount of water molecules (in ppm level) is sufficient to accelerate the unwanted reaction with lithiated phase (since the lithiated phase is highly reactive). Furthermore, the new electrolyte for full cell fabrication will be consumed, which also undergoes the decomposition in the first cycle. As a consequence, the unstable SEI layer ruins the cell performance of full cells upon cycling.^[^ ^1^ ^]^

#### 3.1.2 SC‐EM

To avoid complex operations and large usage of electrolyte caused by HC‐EM, an advanced strategy was proposed to contact the anode directly with Li metal foil as shown in Figure 4B, in which the overall pre‐lithiation can be processed in a simple assembly and the endpoint of the pre‐lithiation is determined by the cell voltage that can be monitored throughout the pre‐lithiation or the pre‐lithiation time that can reflect the pre‐lithiation degree mediately.^[^ ^39^, ^50^, ^51^, ^52^ ^]^ After pre‐lithiation is finished, the lithiated anode can be utilized to match with cathode for full cell assembly.^[^ ^39^ ^]^

In comparison with the HC‐EM, SC‐EM can not only generate SEI on the surface during pre‐lithiation that has very similar characteristics without sacrificing the structural stability of anode, which enables stable cyclability.^[^ ^53^ ^]^ Moreover, the complex deassembling and reassembling separation can be mitigated so that this strategy has the potential to achieve scalable application in a compatible fashion with the conventional roll‐to‐roll battery manufacturing process.^[^ ^54^, ^55^ ^]^

However, the pre‐lithiation speed for SC‐EM cannot be controlled easily as HC‐EM does, which is regulated naturally by the potential difference between anode and Li metal foil. Moreover, there are potential risks of Li metal plating and thermal runaway during the extreme‐fast pre‐lithiation process due to a strong driving force resulted from large potential differences between cathode and anode, as well as low resistance resulted from direct contact structure. Also, the highly reactive lithiated phase will lead to multiple undesirable side‐reactions with a trace amount of water molecules as HC‐EM does, causing capacity degradation upon cycling. Since the anode electrode is wetted by electrolyte during the pre‐lithiation before a full cell assembly so that the battery assembly processes will not be compatible with conventional LIB assembly processes which are needed an ultradry environment.

#### 3.1.3 CM

Alternatively, various chemical methods were proposed in recent years to directly produce the lithiated materials as anode candidates to match with the cathode. The procedures are scalable and the lithiated products are expected to be more stable than those from electrochemical pre‐lithiation strategies as shown in Figure 4C.^[^ ^56^, ^57^, ^58^, ^59^ ^]^

The mechanical alloy (MA) method is proposed, which involves mixing anode materials or precursor (e.g., Si,^[^ ^60^, ^61^ ^]^ SiO,^[^ ^62^ ^]^ SiO_2_^[^ ^62^ ^]^) and Li metal into the ball‐milling machine at the atmosphere of Ar gas at a certain rotary speed.^[^ ^63^, ^64^, ^65^, ^66^ ^]^ In this method, the pre‐lithiation degree can be controlled easily by adjusting the weight ratio of anode materials and Li metal. As demonstrated, the lithiated anode exhibits much higher first CE and much better cycling stability than pristine anode materials, indicating its strong potential as the anode to match with cathode for full cell fabrications.^[^ ^64^ ^]^ In particular, the side‐product of amorphous Li_2_O and/or inorganic Li salt (Li_2_SiO_3_) coating the active lithiated anode can accommodate the volume changes of anode materials, provide a favorable environment for the insertion/extraction of Li ions from particle, and improve the chemical stability of lithiated anode in the surrounding environment.^[^ ^62^, ^67^, ^68^ ^]^

The one‐pot metallurgical method is another method to achieve chemical pre‐lithiation by melting the anode materials and Li metal at about 250–300 °C under mechanical stirring inside a tantalum crucible at a certain speed for several days. As expected, the lithiated anode obtained by this method exhibits improved CE and cycling stability in comparison with pristine anode materials. More importantly, the lithiated anode shows excellent dry‐air stability in a dry room (dew point = −50 ppm) for several days, which is of great significance for the large‐scale productions.^[^ ^60^ ^]^

Although there is no doubt that Li metal is the most suitable reductant for this reduction reaction, owing to its relative light mass, high reactivity toward oxide, and low melting point of Li metal, direct milling of the reactants containing Li metal led the formation of clumps, which hindered the mechanochemical reaction. Besides, the as‐milled product also needs further treatment, such as grinding and sieving for the preparation of the electrodes.^[^ ^69^ ^]^ Therefore, other Li‐containing chemicals (e.g., LiOH and LiH) were proposed as alternative Li source for pre‐lithiation.^[^ ^70^, ^71^ ^]^ The LiOH will transfer into inorganic Li source (e.g., Li_4_SiO_4_) which is unfavorable for the electrode preparation or electrochemical performance.^[^ ^70^ ^]^ The LiH can react with anode materials (e.g., Si) to obtain lithiated anode and the side‐products are hydrogen gas, which will be exposed to air with further treatment.^[^ ^71^ ^]^

The lithiated anode obtained from the abovementioned methods is relatively sensitive to the environment so that it is difficult to prepare electrodes on a large scale based on these materials. To reduce the difficulty of the pre‐lithiation process, an advanced method using liquid Li sources was proposed.^[^ ^72^, ^73^ ^]^ In this method, the electrodes can be prepared in advance and the electrodes are sunk into the liquid Li sources (e.g., Li‐naphthalene in butyl methyl ether) to achieve pre‐lithiation.^[^ ^74^ ^]^ The lithiated anode electrodes were washed with dimethyl carbonate (DMC) to remove the remaining solution and dried for full cell preparation. The pre‐lithiation degree can be controlled by the pre‐lithiation time and the lowest potential is determined by the liquid Li source, for example, 0.21 V for Li‐naphthalene.^[^ ^74^ ^]^

In comparison with HC‐EM and SC‐EM, the process to prepare lithiated anode materials is scalable. However, the electrode preparation using lithiated anode materials is extremely difficult for this method due to the high chemical activity of lithiation anode materials, which has to be carried out using water‐free solvent and in dry atmosphere. Similar to the HC‐EM and SC‐EM, the CM still requires the dry atmosphere to assemble the full cell due to the unstable chemical properties of lithiated anode. Therefore, all the methods to preset the Li source into anode face the challenge of rigorous conditions, which will inevitably increase the manufacturing cost.

### 3.2 Lithium Source in the Cathode

As discussed above, the highly reactive properties of the lithiated anode cannot be addressed effectively so far, disabling the potential commercial application. Therefore, efforts have also been made on presetting the Li source into the cathode, which can mitigate the initial ALL in the charging process of the first cycle (Figure 4D). In this strategy, all the methods can be divided into two main categories, i.e., preparations of over‐lithiated cathode materials with extra Li‐ions (OL‐C) (**Figure** **5**C,D) or adding Li‐containing additive into the cathode (LA‐C) (Figure 5E,F) for mitigating the initial ALL. Some basic parameters need be discussed in particular to evaluate the potential for the future applications, i.e., the Li‐ion supplement capability of the cathode, which determines the usage amount of the Li‐containing materials in cathode; the chemical stability, which is a similar issue as the lithiated anode to tackle toward large‐scale manufacturing; and potential plateau to extract Li‐ions, which should be at the stable range of the electrolyte.

#### 3.2.1 OL‐C

The over‐lithiated cathode (also called “Li reservoir”) refers to material that would deliver extra Li ions to mitigate the initial ALL in the first charge process. In general, the over‐lithiated cathode materials can deliver much more extract Li ions in the first charge as shown in Figure 5B,C, which are stored in the unoccupied crystallographic sites. The typical cathode materials are Li_1+_ *_x_*Mn_2_O_4_, Li_1+_ *_x_*Mn_1.5_Ni_0.5_O_4_, Li_3+_ *_x_*V_2_(PO_4_)_3_, etc., as shown in **Table** **1**.^[^ ^1^, ^75^ ^]^

**Table 1:** The summary of the proposed over‐lithiated cathode and Li‐containing additive in cathode

	^Reference^	Lithium source	Charge plateau [V]	Discharge plateau [V]	Supplied Li capacity [mAh g^−1^]
Over‐lithiated cathode	^[^ ^82^ ^]^	Li_1.144_Ni_0.136_Co_0.136_Mn_0.544_O_2_	4.6	–	48
	^[^ ^83^ ^]^	Li_1+_ *_x_*Ni_0.5_Mn_1.5_O_4_	2.8	2.6	174
	^[^ ^84^ ^]^	LiMn_1.5_Ni_0.5_O_4_	3.8	–	160
	^[^ ^85^ ^]^	Li_1+_ *_x_*Ni_0.5_Mn_1.5_O_4_	2.8	2.7	150
	^[^ ^75^ ^]^	Li_5_V_2_(PO_4_)_3_	1.75	–	400
	^[^ ^81^ ^]^	Li_2_Mn_2_O_4_	4.0	–	30
Li‐containing additive in cathode	^[^ ^90^ ^]^	Al_2_O_3_ coating Li_2_NiO_2_	3.7	1.75	300
	^[^ ^91^ ^]^	Li_6_CoO_4_	3.4	–	350
	^[^ ^97^ ^]^	M‐Li_2_O	3.2/4.0	–	650
	^[^ ^98^ ^]^	M‐LiF	3.2/4.0	1.0	550
	^[^ ^99^ ^]^	M‐Li_2_S	2.5/3.75	–	700
	^[^ ^93^ ^]^	Li_2_S/KB/PVP	3.0	–	1053
	^[^ ^94^ ^]^	Li_3_N	1.0	–	1400
	^[^ ^95^ ^]^	Li_3_N	1.2	–	1800
	^[^ ^96^ ^]^	Li_2_O	4.5	–	1000

Li_1+_ *_x_*Mn_2_O_4_ was first proposed as Li reservoir compounds, providing an excess of Li to the rocking chair cell that will be used at a first charge to overcome the loss of capacity of the anode.^[^ ^1^, ^76^, ^77^, ^78^, ^79^, ^80^, ^81^ ^]^ Li_1+_ *_x_*Mn_2_O_4_ used to be prepared with n‐Butyl‐ Li (n‐BuLi), which needs to be carried in a controlled atmosphere (dry box) and the resulting compound has been demonstrated to be highly hygroscopic. An inherent problem in the use of n‐BuLi is the risk of the irreversible reduction occurring because of its strong reducing power (*V*_n‐BuLi_ = 1 V vs Li/Li^+^) for compounds having electrochemical potentials concerning Li intercalation/insertion. Alternatively, LiI was selected as a milder reducing agent, which can simply synthesize the Li_2_Mn_2_O_4_ at 82 °C in an ambient atmosphere and the obtained Li_2_Mn_2_O_4_ appeared to be air‐stable with strong potential for future applications. Other chemical lithiation processes have also been proposed, such as Li‐alkoxide formation in alcohol by dissolving Li hydroxide or Li metal, respectively, in ethanol or pentanol, which can only react at the temperature lower than ≈80 °C and the obtained Li_1+_ *_x_*Mn_2_O_4_ is prone to lose its Li in ethanol over long reaction period.

**Figure 6:**
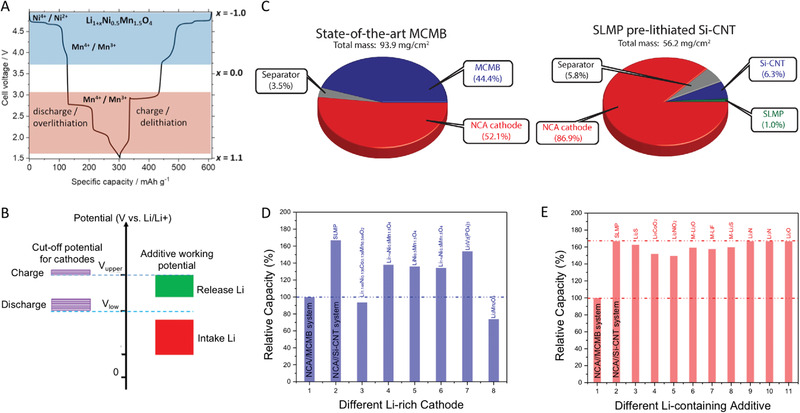
The principle requirements of the A) over‐lithiated cathode and B) Li‐containing additive in cathode for pre‐lithiation. Reproduced with permission.^[^ ^98^ ^]^ Copyright 2016, American Chemical Society. C) The representative mass distribution in a traditional battery and a Si‐based battery. Reproduced with permission.^[^ ^111^ ^]^ Copyright 2013, American Chemical Society. The effect of various D) over‐lithiation cathodes and E) Li‐containing additive in cathode to the relative capacities of Si‐based battery.

Li_1+_ *_x_*Mn_1.5_Ni_0.5_O_4_ is a new type of Li reservoir compound, which can store the extra Li‐ions at the potential lower than 3 V as shown in **Figure** **6**A.^[^ ^82^, ^83^, ^84^, ^85^, ^86^, ^87^, ^88^, ^89^ ^]^ The number of extra Li‐ions can be determined by the potentials after lithiation. To date, there are several methods to prepare Li_1+_ *_x_*Mn_1.5_Ni_0.5_O_4_ using LiI, LiOH·H_2_O, and Li metal as Li sources, respectively. In general, all of these approaches require an additional synthesis step to get to the high Li content without the formation of the unwanted second crystalline phases. Expect for using Li metal as Li source, none of the above strategies is commercially viable, as the necessary components are too expensive, or the synthesis is difficult to scale up. Recently, based on the Li metal as Li source, a simple and scalable synthesis method for Li_1+_ *_x_*Mn_1.5_Ni_0.5_O_4_ are evaluated, in which the commercial LiMn_1.5_Ni_0.5_O_4_ is lithiated in a single step with Li metal in 1‐pentanol and the lithiation degrees are easy to control by adjusting the molar ratio of Li and LiMn_1.5_Ni_0.5_O_4_.^[^ ^84^ ^]^ More impressively, the over‐lithiated cathode materials are chemically stable in ambient air for at least 24 h and can be further processed with organic solvents following the established experimental procedure, which exhibits strong potential for future practical applications.

Recently, Li_5_V_2_(PO_4_)_3_ is proposed as a novel cathode material and extra Li donor as shown in Figure 5B,C.^[^ ^75^ ^]^ Two Li per formula unit is given to the Li_3_V_2_(PO_4_)_3_ and form Li_5_V_2_(PO_4_)_3_ by the electrochemical method using half‐cell configuration, and then the Li_5_V_2_(PO_4_)_3_ can be directly used as cathode materials to lithiated the anode and turn to Li_3_V_2_(PO_4_)_3_ as well. In this method, the lithiation degree can be readily controlled based on the potential after pre‐lithiation. However, the Li_5_V_2_(PO_4_)_3_ shows quiet low stability in air condition. After exposure in the air for 1 day, the lithiated Li_5_V_2_(PO_4_)_3_ structure turns more similar to Li_3_V_2_(PO_4_)_3_ structure for the oxidation of V(II). Thus, lithiated Li_5_V_2_(PO_4_)_3_ can only be stored in an inert atmosphere, which hinders its practical applications.

As discussed above, the OL‐C can supply extra Li‐ions by itself to mitigate the initial ALL in the first charge process, i.e., no extra materials (weight) would be added into cathode to reduce the energy density of devices. However, the capacity of OL‐C to supply Li is relatively low to fully mitigate the initial ALL of the anode, which limits its application fields in LIBs. It is desirable yet challenging to develop novel materials with higher Li supplement capabilities.

#### 3.2.2 LA‐C

An alternative strategy to mitigate the initial ALL is utilizing LA‐C as Li sources as shown in Figure 5E,F. In general, the LA‐Cs need to meet several characteristics. First, a good cathode additive should possess a much higher Li storage capacity by weight and volume than the existing cathode materials. Second, the additive should be able to release its stored Li‐ions below the maximum potential during charge, but not absorb Li‐ions at the minimum potential of cathode discharge. That is, the de‐lithiation potential of additives must be below the maximum cathode charge potential, while the lithiation potential of additives must be below the minimum cathode discharge potential as shown in Figure 6B. This implies a large hysteresis in the delithiation/lithiation potential curve. Third, the cathode pre‐lithiation additive should not have negative effects on the stability of electrode materials, electrolytes, and the whole battery. Usually, that means a relatively high open‐circuit voltage (OCV) is needed. Fourth, the cathode pre‐lithiation additives should ideally be stable in ambient conditions and compatible with existing industrial battery fabrication processes such as slurry mixing, coating, and baking.

Al_2_O_3_‐coated Li_2_NiO_2_ (A‐L2NO) was early proposed as LA‐C material, which has a delithiation potential at ≈3.6–4.0 V and two‐step lithiation potential of ≈4.0–3.5 and ≈2.0–1.5 V.^[^ ^91^ ^]^ This material is theoretically suitable for the application in the system using LiCoO_2_ as cathode and graphite as the anode (operating voltage range of ≈2.8–4.0 V), in which A‐L2NO can supply the Li‐ions about 200 mAh g^−1^. The additive after the first charge still delivers a reversible capacity of about 100 mAh g^−1^ to make up the capacity of the systems to a certain degree. Moreover, the coating of Al_2_O_3_ has largely improved the stability of L2NO. After exposure to air for 7 h, only a small amount of powder leads to the formation of nonconducting Li_2_CO_3_, and LiOH phases on the particle surface with no obvious capacity decrease. Overall, A‐L2NO can meet the requirements as an LA‐C material in general but the low capacity restricts the applications to those systems with relatively low initial ALL, rather than next‐generation practical LIBs with high initial ALL.

To enhance the capacity, more Li metal oxides as LA‐C materials were developed in recent years. Antifluorite Li_6_CoO_2_ is a promising LA‐C material with the capability to supply the Li‐ions about 350 mAh g^−1^ at the potential range of ≈1.0–4.75 V for the applications in the LiCoO_2_ system.^[^ ^91^ ^]^ Due to its high capacity, this material achieved pre‐lithiation for the SiO‐based anode.

Sacrificial salts are types of Li donors in the cathode. Different from the above Li sources, the product after the first charge is gas which can be eliminated from cells after the formation process. There are a series of sacrificial salts proposed for LIBs, e.g., azide (LiN_3_), squarate (Li_2_C_4_O_4_), oxalate (Li_2_C_2_O_4_), ketomalonate (Li_2_C_3_O_5_), and di‐ketosuccinate (Li_2_C_4_O_6_), which would transform into gases such as N_2_, CO, or CO_2_ at the potential range of ≈3.0–4.5 V.^[^ ^92^ ^]^ However, these salts are usually insulating so that a large portion of conductive agents is necessary for the electrode preparation. Moreover, the transformation from solid salts to gas would destroy the electrode structure which hinders the practical applications.

Li*_x_*Y (Y = O, N, S) type materials are considered as the most ideal LA‐C materials with ultrahigh capacity over 1000 mAh g^−1^. However, this type of material has a relatively high activated energy so that more special treatments are necessary. Core–shell Li_2_S/KB/PVP was developed as Li donor in the LiFePO_4_ system (with a potential range of ≈2.5–3.8 V).^[^ ^94^ ^]^ The improved electric conductivity and the good compatibility with carbonate electrolyte enable this composite to supply a high capacity of about 1050 mAh g^−1^. However, the formed insoluble and insulating sulfur after the first charge will cover the surface of active materials and thus reduce the electrochemical performance. Li_3_N and Li_2_O were also demonstrated as effective donors in the LiCoO_2_ systems.^[^ ^94^, ^95^, ^96^ ^]^ Different from Li_2_S, these materials are converted to nitrogen or oxygen gas after the first charge, which would result in a structure collapse of electrodes and thus limits the practical application. Moreover, this type of material has a common challenge due to high chemical reactivity with moisture in the ambient atmosphere which also hinders the practical applications.

Recently, Sun et al. proposed a type of nanosized composite of M‐Li*_x_*Y (M = Fe, Co, Ni, Mn, etc., Y = O, S, F) by chemical reaction of MY and metal Li, which enables to deliver a high capacity of 500–1000 mAh g^−1^.^[^ ^97^, ^98^, ^99^ ^]^ The introduction of metal in this system has several advantages: first, the lithi Li action mechanism changes from a decomposition reaction to a conversion reaction, which is particularly significant for the M‐Li_2_S system to address the issue of incompatibility of electrolytes for Li_2_S; Second, the electrochemical Li and delithiation potentials are reduced leading to the full extraction of Li from the additive below the cutoff charge potential, while the low lithiation potential of MY ensures that the Li does not transform back to the initial state above the cutoff discharge potential. In other words, due to the potential limit, the conversion reaction of M‐Li*_x_*Y composites is nonreversible for extracting Li. Thus, as a cathode pre‐lithiation additive, the donor Li‐ion capacity of the M‐Li*_x_*Y composite can be maximized; third, the metal in the composite and as‐formed MY during conversion reactions can immobile the polysulfide intermediates and prevent their irreversible reaction with carbonate electrolyte for sulfur‐based materials, and there is no formation of sulfur, oxygen, or fluorine in this process for enhanced integrity of the electrode; last, the as‐designed M‐Li*_x_*Y nanocomposites have better ambient stability than that of pristine Li*_x_*Y particle, which paves the way for practical applications.

Therefore, in comparison with OL‐C, the LA‐C has a relatively higher capacity to mitigate the ALL of the anode, which is favorable to preset the Li source into the cathode. However, some Li sources after releasing Li‐ions would become dead and sometimes insulating materials in the cathode, which possesses negative effects on the specific capacities of the systems. To illustrate the effects quantitatively, we tried the estimated energy density calculation only based on the weight of cathode, anode, separator, and Li source for the Lithium Nickel Cobalt Aluminum Oxide (NCA)/ Silicon‐carbon nanotube (Si‐CNT) system as shown in Figure 6C. In comparison with the NCA/mesocarbon microbeads (MCMB) system, the NCA/Si‐CNT system with pre‐lithiation has the capability of higher capacity output. As expected, the capacity of Li sources has a big influence on the capacity of the full cell as shown in Figure 6D,E. In detail, the LA‐C could achieve a higher capacity of the full cell than OL‐C due to their higher capacity as shown in Table 1. Most importantly, an unexpected result can be obtained that the influence of the dead materials of the LA‐C on the capacity of the cell is relatively acceptable. The obtained capacity of the NCA/Si‐CNT system is still 50% higher than NCA‐MCMB at least (Figure 6E). In particular, using the Li_3_N and Li_2_O as Li sources, there are no dead materials after pre‐lithiation. These results mean the strategy of Li sources in the cathode is exactly promising for the next generation LIBs even if some dead materials have to be introduced into the system. However, there are also some challenges developing an ideal Li source for pre‐lithiation. Some Li sources will generate gases (e.g., N_2_, O_2_, etc.) after the pre‐lithiation process, which may destroy the structure of electrodes. Moreover, most of the current Li sources are still not stable in ambient conditions, which would bring complex operation and extra cost.

### 3.3 Sacrificed Electrode Method

As discussed above, both the lithiated cathode and anode still suffer from chemical instability, which hinders the practical applications. Therefore, several attempts have been made to add Li sources into the system when fabricating the cells. Inspired by the pre‐lithiation technique developed by JM Energy in Japan, which enables a full pre‐lithiation of carbon anodes (i.e., to form the LiC_6_ state at the graphite anode) in Li‐ion capacitors (LICs) to lower the electrode potential of carbon anode, an improved pre‐lithiation of graphite anodes using through‐holed cathode and anode electrodes in a laminated LIB was proposed.^[^ ^100^ ^]^

**Figure 7:**
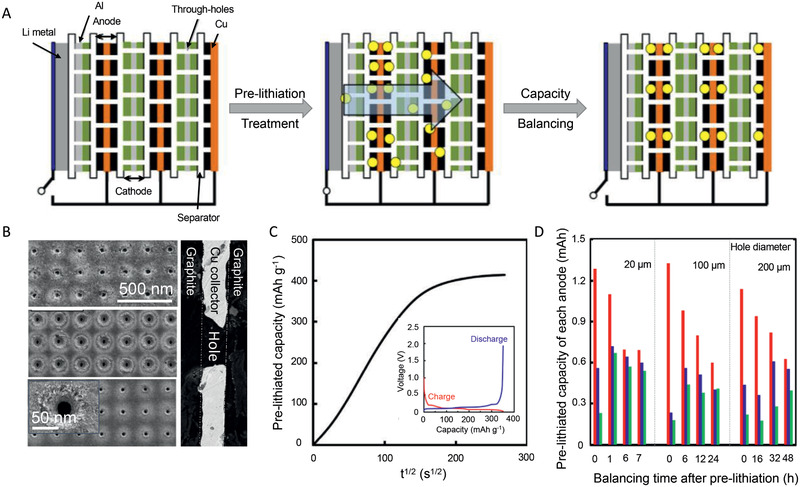
A) The schematic graph of sacrificed electrode method; B) The SEM graphs of electrodes with holes for ion diffusion. C) The relationship between pre‐lithiated capacity to the time (inserted graph is the charge and discharge profiles of pre‐lithiated graphite). D) The relationship between the pre‐lithiation degree on every electrode and balancing time after pre‐lithiation. Reproduced with permission.^[^ ^100^ ^]^ Copyright 2019, Elsevier Ltd.

In this method, un‐lithiated anodes, cathodes, and separators are laminated in a cell and then a Li metal foil is set to the assembly. When Li metal is connected to the un‐lithiated anodes, the pre‐lithiation to the anodes starts. To fully pre‐lithiate Li ions to all the anodes in the cell, all of the anode and cathode in the cell have through‐holed current collectors and consequently, Li‐ions can pass through them gradually during the pre‐lithiation process as shown in **Figure** **7**A. This technique is referred here as a “perpendicular” Li pre‐lithiation method based on JM energy's original designation in Japanese because the direction of the movement of Li ions is perpendicular to the laminated anode and cathode surface. Note that the through‐holes with the average hole diameter of 20 µm are formed directly on the cathode and anode with a pico‐second pulse laser system. As demonstrated, the pre‐lithiation rate significantly depends on the hole diameter and hole opening rate, which is effectively increased by decreasing the hole diameter from 200 to 20 µm at a given hole opening rate (Figure 7B). Besides, the potentials of the cathode and anode during pre‐lithiation was monitored concerning Li metal reference which was located next to the assembly of anodes, cathodes, Li metal, and separators. The pre‐lithiation process would decrease the potential of anode due to the electrochemical process with Li metal (Figure 7C), but there is not any effect on the cathode. Even for a pre‐lithiation period of 80 h, the cathodes still showed a stable potential as the original state.

As a result, the sacrificed electrode method is a relatively practical for the pre‐lithiation, in which the lithiation degree can be well controlled by the potential of the anode, the integrity of electrodes can be maintained in the overall pre‐lithiation process, and only one procedure of presetting Li metal foil needs to be carried out in the dry room. However, there are still some issues to resolve: the pre‐lithiation efficiency is low, i.e., a long period is required for the full pre‐lithiation, and the electrodes with through‐holes significantly increase the cost of the cells. As shown in Figure 7D, the lithiation degree in various electrodes is not uniform and thus a long time is necessary for the balance of the lithium degree.

### 3.4 Extra Lithium Source

**Figure 8:**
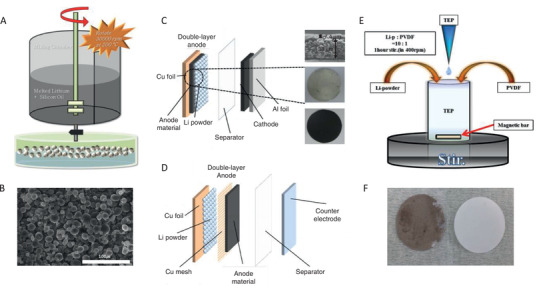
A) The preparation scheme and B) SEM graph of stabilized lithium metal powder (SLMP). Reproduced with permission.^[^ ^114^ ^]^ Copyright, 2014, IOP Publishing. C) Two types of structures with SLMP for pre‐lithiation. Reproduced with permission.^[^ ^101^ ^]^ Copyright, 2016, IEEE. D) Reproduced with permission.^[^ ^113^ ^]^ Copyright, 2010, Elsevier. E) The scheme of the coating of SLMP onto separator. F) The comparison of SLMP coating separator and bare separator. Reproduced with permission.^[^ ^114^ ^]^ Copyright, 2014, IOP Publishing.

In this strategy, special Li metal source or structures for anode were carefully designed to enable high‐efficiency pre‐lithiation without the usage of through‐holed electrodes. The stabilized Li metal powder (SLMP), a commercial product from the FMC Corporation (USA), is a special Li source with a particle size of 10–20 µm that can be safely handled in a dry‐air atmosphere (**Figure** **8**A,B).^[^ ^101^ ^]^ The improved dry‐air stability of SLMP is attributed to a protective layer of Li_2_CO_3_ around the Li particles, and therefore a certain pressure to break up the Li_2_CO_3_ coating is required for activating the SLMP.^[^ ^102^ ^]^

**Figure 9:**
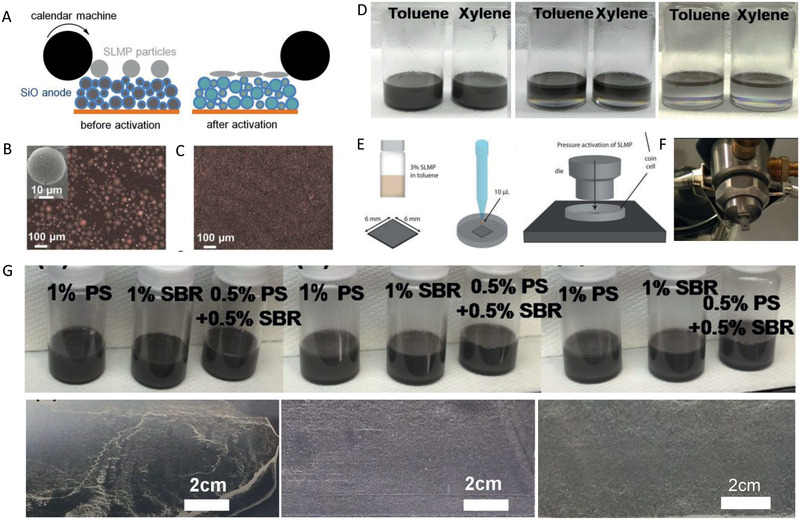
A) The scheme of simply coating of SLMP on the electrode. The SEM images of electrode with SLMP B) before and C) after pressure activation. Reproduced with permission.^[^ ^111^ ^]^ Copyright 2013, American Chemical Society. D) The standing of the SLMP in Toluene and Xylene with time. E) The scheme of drop‐casting method. Reproduced with permission.^[^ ^111^ ^]^ Copyright 2013, American Chemical Society. F) The tool for practical air‐brushing method. G) solution‐processed slot dye coating method. Reproduced with permission.^[^ ^103^ ^]^ Copyright 2016, Elsevier.

In the beginning, the SLMP was simply coated onto the electrode as Li source as shown in Figure 8C.^[^ ^39^, ^103^, ^104^, ^105^, ^106^, ^107^, ^108^, ^109^ ^]^ After soaking with electrolyte, the Li metal is transferred to Li ions and the Li ions in electrolyte were injected into electrodes for pre‐lithiation. Due to the inactive Li_2_CO_3_ coating, the reaction between SLMP and electrolyte is minimized. SLMP can be used as a Li source to achieve pre‐lithiation after pressure activation (**Figure** **9**A). However, due to the small particles, it is quite difficult to achieve a uniform dispersion on the electrode during the cell fabrication process at a practical level (Figure 9B,C), which motivates researchers to find a better way of incorporating the SLMP into the cell with reduced manufacturing cost.

The slurry method was first proposed based on the Styrene Butadiene Rubber‐Polyvinylidene fluoride (SBR‐PVDF) binder.^[^ ^110^ ^]^ Due to the incapability with polar solvents, e.g., N‐methyl pyrrolidinone (NMP), dimethylacetamide (DMA), and dimethylformamide (DMF), the active materials were coated by PVDF first to form the composite, which was then mixed with SLMP, carbon black, and SBR in toluene or hexane. The SLMP was then successfully preset in the electrodes obtained from this special slurry. However, in this method, all the electrodes preparation processes should be carried out in a dry‐air atmosphere, which inevitably increases the costs of manufacturing.

The drop‐casting method achieves the SLMP coating after electrode preparation processes (Figure 9D). The SLMP was added to toluene to form an SLMP/toluene suspension at a 3% SLMP w/w loading.^[^ ^111^ ^]^ Because of the low density of pure Li metal (0.53 g mL^−1^), the SLMP particles (containing 97% Li) rise to the top of the toluene, necessitating a small stir bar to keep the SLMP mixed in the toluene during SLMP deposition. The suspension was dropped‐cast on the surface of the electrode directly. After the toluene was evaporated, the SLMP coating electrodes were obtained. The pressure‐activation step was conducted in a dry state before adding electrolytes to the cell, which ensures proper Li‐ion diffusion into the anode and increases the consistency among batteries for the present case. In this method, only the SLMP drop‐casting process and pressure‐activation step are carried in a dry state, largely decreasing the manufacturing cost.

Based on the drop‐casting method, a more practical method for SLMP coating on the electrodes, i.e., the air‐brushing method, was proposed (Figure 9E).^[^ ^112^ ^]^ The suspension of SLMP in toluene was homogeneously applied on the surface of the electrode with a commercial airbrush, which needs to be carried out in a dry atmosphere. When toluene was evaporated, the powder was roll pressed onto the surface of the electrodes with a pestle manually. By doing that, the dull surface of the electrode turned into a shiny metal‐like one, indicating that the protective layer of SLMP was broken, and the SLMP was well dispersed on the surface of the electrode. This method enables a more homogeneous dispersion of SLMP on the surface of the electrode.

As mentioned above, the SLMP always rises to the top of the toluene solvent, which makes it difficult to achieve homogeneous dispersion of SLPM on the surface of the electrode (Figure 9G).^[^ ^13^ ^]^ To overcome this drawback, a solution‐processed slot dye coating method was proposed for uniform and scalable SLMP coating. The SLMP was added into a binder solution, which was made by dissolving SBR and carbon black well in xylene. The loading amount of SLMP can be controlled by varying the amount of SLMP in solution and the gap for the doctor blade. After the xylene is dried off, the electrode with SLMP coating is calendared with a rolling press to activate the SLMP. This method enables uniform and scalable SLMP coating methods and the manufacturing coat is not as high as the slurry method due to the thin coating layer.

On the other hand, to well preset the Li sources, some special electrode structures are designed and evaluated (Figure 8D).^[^ ^113^ ^]^ Double layer anodes were prepared using an SLMP layer electrode and an anode electrode, which were separated by a Cu mesh. The Li powder was coated onto the Cu foil by the tape casting method and the obtained electrode was positioned on the backside of the anode electrode, which was prepared by the doctor blade method using Cu mesh as the current collector. This method can also achieve satisfactory pre‐lithiation, but the usage of Cu mesh and the consumption of Li powder will increase coat significantly.

Interestingly, the SLMP was also attempted to be coated onto the separator (Figure 8E).^[^ ^114^, ^115^ ^]^ In comparison with the coating process on the electrodes, the coating on the separator can be easily handled with precise control of quantity. The SLMP and PVDF were dissolved in a triethyl phosphate (TEP) solvent first at a certain weight ratio. Then, a single side of the prepared separator (PP) was dipped into the slurry and dried for 24 h in a dry atmosphere. This represents a simple method for SLMP coating with low manufacturing costs.

To summarize, the method to use extra Li sources is considered a promising pre‐lithiation on a practical level. However, there are still some challenges: i) the dispersion issue of SLMP has not to be addressed although several methods are proposed to remit the precipitation; ii) the method is relatively risky to cause short circuit due to the relatively low purity of SLMP (<98%); iii) the SLMP with small particle size is difficult to control and has the hidden danger for the explosion; iv) compared with SLMP, the ultrathin Li film is a better alternative but is still impractical for large‐scale production.

### 3.5 Comparisons of Various Representative Pre‐Lithiation Strategies

**Figure 10:**
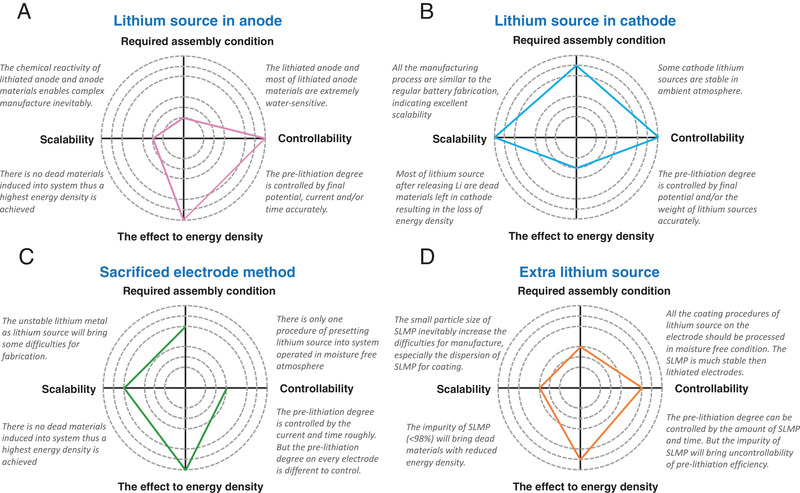
The detailed characteristics comparison for the strategies of A) lithium source in anode. B) Lithium source in cathode. C) Sacrificed electrode method. D) Extra lithium source in the fields of required assembly condition, controllability, the effect to energy density, scalability. Note: The outer circles are better than inner circles.

In this section, four types of pre‐lithiation strategies, i.e., Li source in anode, Li source in the cathode, sacrificed electrode method, and extra Li source, have been carefully summarized. Here, the main properties of required assembly condition, controllability, scalability, and the effect on the energy density of full cells for these pre‐lithiation strategies are carefully compared in **Figure** **10**. In general, all the current pre‐lithiation strategies still face different challenges.

Li source in the anode is suitable for the applications in lab studies due to its excellent controllability and no negative effect on the energy density of full cells. The lithiated anode and/or anode materials are extremely water‐sensitive so that it requires strict assembly condition for all the fabrication processes, and it is difficult to achieve large‐scale applications. However, if the water sensitivities of chemical lithiated materials can be addressed, the strategies of Li source in anode still have the potential for commercial applications.

Li source in the cathode is a promising pre‐lithiation strategy with desirable properties for fabrication in the ambient atmosphere, as well as good controllability and scalability. However, the dead materials are left after releasing the Li from the Li source (e.g., Li_6_CoO_4_, LiMoO_3_, Li_2_S, etc.), which leads to reduced energy density inevitably. Although some novel Li sources (e.g., Li_3_N, Li_2_O, etc.) were developed whose products after pre‐lithiation are pure gas, the gas formation would destroy the integrity of the electrodes. Moreover, this type of Li source is still water‐sensitive, which would increase the difficulties for fabrication.

The sacrificed electrode method is already a practical strategy in the field of the Li‐ion capacitor (LIC), but it has not been widely applied in LIBs. In this method, only the process to preset the Li source into cells needs to be carried out in a moisture‐free atmosphere, which is acceptable for production. However, the difficulties for this method are the controllability of the pre‐lithiation degree and rate. The pre‐lithiation degree can be controlled by the potential, pre‐lithiation time, and the amount of Li source in the cell, but the observation is in cell level instead of a single electrode level. The imperfect uniformity of the pre‐lithiation degree on the anodes could bring the risks of Li dendrite growth and even thermal runaway. Therefore, more studies need to be carried on to precisely control the pre‐lithiation condition for more stable performance.

Extra Li source is another practical strategy in the field of LIC. In comparison with the sacrificed electrode method, both procedures of coating Li sources and battery assembly should be processed in a moisture‐free condition. Moreover, Li sources need to be coated onto every electrode which reduces the scalability to a large degree. However, this strategy increases the controllability of the pre‐lithiation as the pre‐lithiation for each electrode is uniform. Currently, the Li source for this method is SLMP, which has a small particle size and relatively low purity (<98%), bringing about a negative effect on the energy density of full cell and difficulties for uniform dispersion on the electrode surface. On the other hand, the small size of SLMP also induces the risk of explosion. Therefore, it is desirable to develop ultra‐thin Li foil to replace the SLMP for easier coating and safer operation, which is not available in the market yet due to production difficulty.

## 4 The Representative Applications of Current Pre‐Lithiation Strategies

### 4.1 The Applications of Pre‐Lithiation Strategies in Si‐Based Systems

Si is a promising anode active material due to its extremely high specific capacity (4200 mAh g^−1^) and low potential, but the primary disadvantage is that the large volume changes (>400%) can lead to rapid pulverization of Si particles and loss of capacity during cycling. A common approach to improving the cycling performance of Si‐based anodes is to use nanostructured Si. However, the high surface area of nanostructured materials significantly increases SEI formation in the first cycle. SEI formation on Si anodes during the first cycle causes a high ALL and can result in low CE values, such as 25–80%, depending on the structure of the Si and the composition of the anode composite (Figure 3A). Therefore, pre‐lithiation technologies have been widely used in Si‐based systems.

As discussed above, the cathode pre‐lithiation strategies are useful but the capacity supply is relatively limited, by which the energy density of full cells would be sacrificed. Therefore, there are only a few researches to achieve pre‐lithiation for Si‐based systems using cathode pre‐lithiation strategies. Moreover, in this literature, the Li source used is the highest in the all proposed Li sources for cathode pre‐lithiation, while the reported Si‐based anode is of relatively low ALL. For most of Si‐based systems, HC‐EM, preparing Li‐Si alloy, and adding extra Li sources are dominating pre‐lithiation strategies to fully supply the number of Li sources to mitigate the initial ALL (Figure 5A).

HC‐EM are applied in the lab studies and the characteristics of formed SEI will affect the performance of lithiated anode. Compared with the initial discharge capacity of SiO*_x_* using normal discharge with constant current density (ND), lower initial discharge capacity and higher CE by short‐circuit‐containing constant‐resistance (PLSC) were observed, and the PLSC is capable to form the SEI around the surface of the particles in the electrode.^[^ ^53^ ^]^ The SEI formation using PLSC enables better Li‐ion diffusion for improved capacity and high‐rate performance. To form the SEI well by PLSC, various SiO*_x_* electrodes were prepared using a controlled pre‐lithiation process that used an optimized circuit resistance for delicate control of the lithiation. During the initial discharge, it was verified that not only the excess formation of the SEI but also the insufficient formation of SEI results in poor electrochemical performance. This finding indicates that a suitable amount of SEI in the electrode can be used to prepare anode materials with optimal properties for LIBs. In particular, the effect of the SEI on the electrochemical performance over the entire cycling process was mainly determined by the initial formation of the SEI in the electrode.

**Figure 11:**
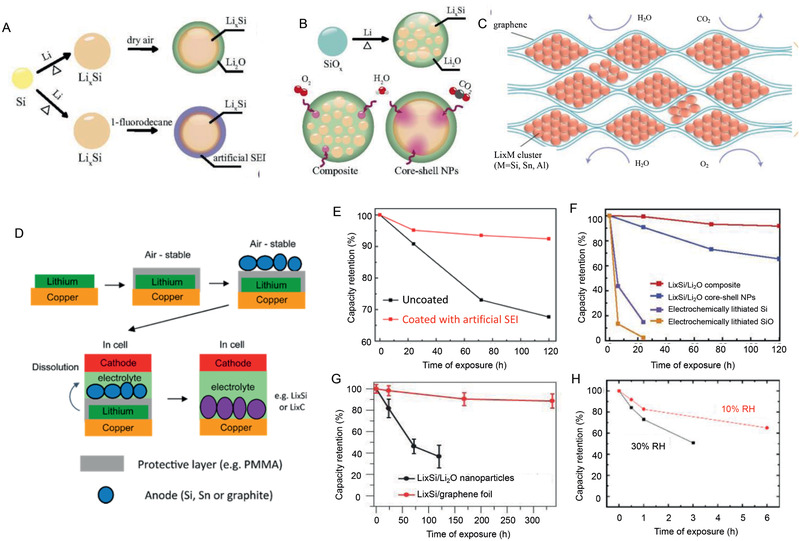
The schematic graphs of A) Li*_x_*Si/Li_2_O core–shell nanoparticles and artificial SEI coating Li*_x_*Si nanoparticles. B) Li*_x_*Si/Li_2_O campsite. C) Free‐standing Li*_x_*M/graphene foil (M = Si, Sn, or Al). Reproduced with permission.^[^ ^3^ ^]^ Copyright, 2017, Nature Publishing Group. D) Special electrode design to prepare an ambient‐air stable lithiated anode with uniform distribution of lithium source. Reproduced with permission.^[^ ^119^ ^]^ Copyright, 2016, American Chemical Society. E–H) The capacity retention after exposure to ambient air for different composites. Reproduced with permission.^[^ ^117^ ^]^ Copyright 2015, American Chemical Society.

In recent decades, a series of Li–Si alloy materials were developed, i.e., Li_21_Si_5_ and Li_17_Si_4_, Li_15_Si_4_, Li_13_Si_4_, Li_12_Si_7_, Li_7_Si_3_, LiSi, using MA method, one‐pot metallurgical method, etc.^[^ ^60^, ^61^, ^62^, ^63^, ^64^, ^65^, ^67^, ^69^, ^70^, ^71^, ^74^, ^116^ ^]^ However, the surface texture of these Li–Si alloy materials change after the air exposure for several hours. An air‐stable, Li‐containing anode with long‐term cycling stability and high CE is therefore highly desired. During the recent several years, some nanomaterials engineering methods and special structural designs were proposed to improve stability. To enable stability in dry air and a low‐humidity environment, the Li*_x_*Si nanoparticles were exposed to trace amounts of dry air, resulting in the formation of a Li_2_O passivation layer (**Figure** **11**A). The Li*_x_*Si/Li_2_O core–shell nanoparticles maintained their capacities only in the dry air, while their capacities were reduced drastically after exposure to ambient air (Figure 11F). In the following study, an artificial SEI consisting of LiF and Li alkyl carbonate with long hydrophobic carbon chain coating Li*_x_*Si nanoparticles were prepared using 1‐fluorodecane (Figure 11A).^[^ ^117^ ^]^ The coated Li*_x_*Si nanoparticles showed improved air stability only at a low‐humidity level (<10% relative humidity (RH)). It is still difficult to realize perfect encapsulation and that any pinhole will provide a pathway for inner Li*_x_*Si to react with water vapor in the air to significantly reduce the capacity (Figure 11E). Next, the Li*_x_*Si/Li_2_O campsite with excellent ambient air compatibility through a one‐pot metallurgical process using low‐cost SiO or SiO_2_ as the original material to alloy thermally with molten Li metal (Figure 11B).^[^ ^118^ ^]^ The composite revealed a unique structure with homogeneously dispersed active Li*_x_*Si nanodomains embedded in a robust Li_2_O matrix, which gave the composite unparalleled stability in both dry air and ambient air (≈40% RH) conditions, superior compared with previous developed Li*_x_*Si/Li_2_O core‐shell nanoparticles. That is contributed by a high crystalline Li_2_O matrix formed at high temperature and the enlarged contact area between Li_2_O and Li*_x_*Si, which enables slurry processing of anode using this composite (Figure 11F).

Besides, some special designs are also proposed to improve the stability of the alloy materials (Figure 11C). A facile process to fabricate large‐scale and free‐standing Li*_x_*M/graphene foil (M = Si, Sn, or Al) with the unique nanostructure of densely packed reactive Li*_x_*M nanoparticles encapsulated by large graphene sheets (graphene refers to few‐layer (<10 layers) graphene).^[^ ^3^ ^]^ The excellent air‐stability of Li*_x_*M/graphene foils is attributed to their unique structure where each Li*_x_*M cluster is surrounded and protected by the large graphene sheets. Theoretically, the hydrophobicity and gas impermeability of the graphene sheets prevent the adsorption and penetration of gas molecules. Moreover, the air‐stability can be further improved by the atomic layer deposition of oxides or fluorides on the double sides of the foils (Figure 11G). On the other hand, special electrode design is also proposed to prepare an ambient‐air stable lithiated anode with a uniform distribution of Li source (Figure 11D).^[^ ^119^ ^]^ The electrode is stable in air with RH of 10–30% for over 60 min which allows for manufacturing at large scale (Figure 11H). Moreover, the polymeric protective layer in this design is readily soluble in the electrolyte, and thus no excessive inactive materials, such as protective Li_2_O or Li_2_CO_3_ layer, remain in the battery and reduce battery energy density. In addition, the amount of Li in the anode is easily adjusted by controlling the thickness of the Li layer. The latter case allows the lithiated anode to pair with high capacity Li‐free cathode materials to further improve the energy density of LIBs.

**Figure 12:**
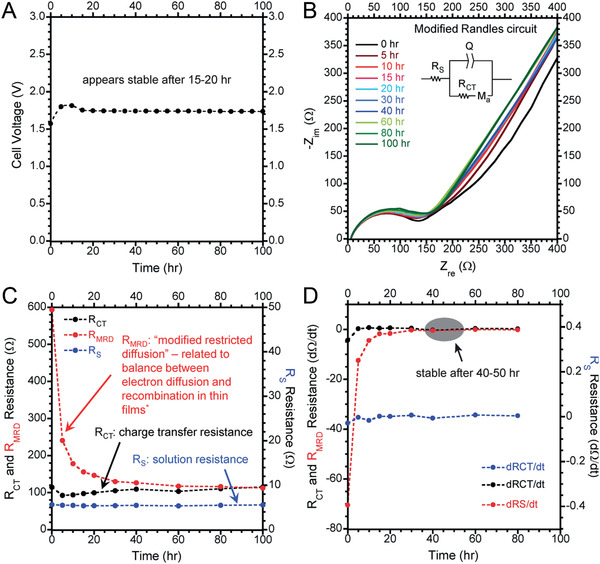
The detailed main parameters of *R*_s_ (solution resistance), *R*_CT_ (charge transfer resistance), and *R*_MRD_ (modified restricted diffusion) were monitored with the pre‐lithiation time, which are used to determine the stable time for pre‐lithiation using SLMP. Reproduced with permission.^[^ ^111^ ^]^ Copyright 2013, American Chemical Society.

Adding extra Li sources is another mainstream strategy for Si anode using the SLMP methods as discussed in Section 3, e.g., slurry method, drop costing method, air‐brushing method, etc. To date, the conditions to achieve the pre‐lithiation have been well illustrated. Without pre‐lithiation, the CE of the first cycle of the composite anode was about 64%; however, the CE increased to 98% after applied SLMP to the surface of the composite anode electrode. When the SLMP and anode mass ratio is 0.26, a reversible specific capacity of 1230 mAh g^−1^, which is much higher than the capacity of commercial graphite anode.^[^ ^120^ ^]^ The effect of pre‐lithiation time was investigated using an NCA/Si‐CNT with SLMP. The main parameters of *R*_s_ (solution resistance), *R*_CT_(charge transfer resistance), and *R*_MRD_ (modified restricted diffusion) were monitored with the pre‐lithiation time. The *R*_s_ is measured to be constant all the time at ≈5.5  Ω. The *R*_CT_ decreases during the first 40 h and restabilizes around 50 h near the original value of 115  Ω. The *R*_MRD_ drops by a factor of 4 during the first 30 h of equilibration time and has eventually stabilized by 40–50 h. As shown in **Figure** **12**, it was confirmed that the fitting parameters have stabilized by 40–50 h, which suggests that it takes ≈40–50 h of equilibration time for the SLMP to fully prelithiate this type of Si‐CNT anode through Li diffusion and any spontaneous SEI formation. Next, the effect of SLMP with/without pressure activation was also demonstrated. The charge profiles for the drop‐cast SLMP and anode without SLMP show a pronounced sloping voltage plateau during the first 0.5 mAh of charge, which is attributed to SEI formation on the Si and CNT current collector. The SEI formation will cause ALL and the presence of such a voltage plateau indicates that a Si‐CNT anode has not been properly pre‐lithiated. When a Si‐CNT has been properly pre‐lithiated, the voltage profile from SEI formation is not present, as shown for the p‐SLMP. As a result, the cell delivers a wider distribution of discharge capacity and stable cycling performance when properly applying and pressure‐activating the SLMP during fabrication, as well as equilibrating for 40–50 h after fabrication. Also, the capacity ratio of SLMP and anode, and the electrodes matching of cathode and anode are demonstrated in the published studies.

### 4.2 The Applications of Pre‐Lithiation Strategies in Lithium‐Ion Sulfur Batteries

In recent years, some novel Li‐free cathode materials (e.g., S, V_2_O_5_) with high capacity were proposed for high energy LIBs.^[^ ^121^, ^122^, ^123^ ^]^ Sulfur, with a high theoretical specific capacity of 1672 mAh g^−1^ (based on the reaction: S_8_ + 16 Li^+^ + 16 e^−^ = 8 Li_2_S), low price)0.1 $ kg^−1^(, and environmentally friendliness, is considered as the most promising cathode material candidate. However, the lack of Li‐ion in the S cathodes inevitably requires Li metal as the anode, which raises several issues for commercialization. The uneven deposition on Li anode during cycling caused by ununiform SEI will result in the continuous growth of Li dendrite, leading to internal short and easily triggering safety issues like explosion or burst especially in enlarged scale of Li–S battery (LSB) in practical application. Li‐ion sulfur battery is one of the most promising solutions to tackle the hazard triggered by Li metal. In recent years, encouraging progress has been made in making LSB systems by applying pre‐lithiated electrodes.

**Figure 13:**
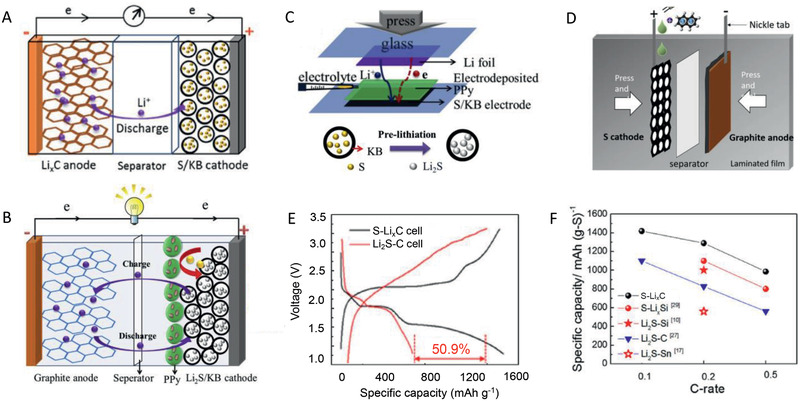
The structures of A) sulfur cathode coupled with lithiated anode and B) lithiated cathode coupled with anode. Reproduced with permission.^[^ ^124^, ^131^ ^]^ Copyright 2016, 2018, Elsevier. C) The direct contact method for cathode pre‐lithiation. Reproduced with permission.^[^ ^124^ ^]^ Copyright 2016, Elsevier. D) The pre‐lithiation using chemical lithium source. Reproduced with permission.^[^ ^128^ ^]^ Copyright 2019, Elsevier. E,F) The performance comparisons for two different structures. Reproduced with permission.^[^ ^129^, ^131^ ^]^ Copyright 2018, 2019, Elsevier.

On one hand, Li‐ion sulfur batteries can be realized by coupling a conventional sulfur cathode with a lithiated anode, which can be prepared with the previous methods (**Figure** **13**A).^[^ ^124^, ^125^, ^126^ ^]^ On the other hand, they can be prepared by coupling a Li_2_S cathode with a Li‐free intercalation/insertion negative electrode material such as graphite and Si (Figure 13B), in which configuration Li_2_S inherently have volume accommodation properties built‐in to mitigate the volume expansion concerns. However, the handling of Li_2_S for large‐scale manufacturing is problematic due to its high moisture sensitivity. Typically, the Li_2_S cathode can be prepared by the direct synthesis of Li_2_S composite materials or pre‐lithiation of a sulfur cathode. The former approach is beyond the scope of this review and is already summarized in some recent review papers. In the latter approach, the pre‐lithiation has been achieved by HC‐EM and DC‐EM method (Figure 13C). Moreover, the chemical lithiation to convert sulfur to Li_2_S shows great promise.^[^ ^127^, ^128^, ^129^ ^]^ The successful pre‐lithiation of the sulfur cathode by dropping a Li‐organic complex solution Li naphthalenide on the prepared sulfur composite electrode at room temperature was also proposed (Figure 13D).^[^ ^130^ ^]^ The further research employed this idea to use Li naphthalenide to fully pre‐lithiate sulfur‐poly(acrylonitrile) (S‐PAN) composite into a Li_2_S‐PAN cathode and to partially pre‐lithiate Si into a Li*_x_*Si anode, which leads to a new version of Li‐ion sulfur battery with specific energy and CE.

It needs to be pointed out that besides their pros and cons for practical operation, the use of sulfur cathode or Li_2_S cathode has one important difference from the pre‐lithiation standpoint of view, namely, for Li_2_S cathode part of Li‐ion has to be used for compensating the Li loss on the anode side during the initial cycles. This has been confirmed as shown in Figure 13E,F that the Li‐ion sulfur cell comprising the pre‐lithiated graphite anode shows a superior performance toward the pre‐lithiated cathode.^[^ ^131^ ^]^

## 5 Perspective and Outlook

In this progress report, the issue of initial ALL for next‐generation LIBs was introduced, which hinders their large‐scale application inevitably. The origin of the huge initial ALL was mainly ascribed into four possible causes, i.e., the formation of solid electrolyte interphase, the loss of active materials, trapped Li‐ion in the host materials, and/or the irreversible electrochemical reactions. The effect of the huge ALL on the energy density of the full cell was also discussed. The effective cell capacity was sacrificed due to the huge ALL, which indicates the effective capacity would recover to full cell capacity only if the initial ALL was compensated by the pre‐lithiation treatment. After that, current pre‐lithiation technologies were summarized, classified, and compared based on the components where the Li sources were preset, i.e., Li source in the anode, Li source in the cathode, sacrificed electrode method and extra Li source. All these strategies exhibit their advantages and disadvantages in the case of required assembly condition, controllability, scalability, and the effect of pre‐lithiation on the energy density of the full cell. In general, all these strategies still face various challenges for practical application. Finally, the representative applications of pre‐lithiation strategies were introduced for the Si‐based battery system and Li‐ion sulfur batteries. This progress report will bring up new insights to reassess the significance of pre‐lithiation strategies for the next generation of LIBs.

On the other hand, we also would like to offer a guideline for the research directions based on the pre‐lithiation strategies summaries and comparisons. As current pre‐lithiation strategies possess several bottlenecks for the practical applications, we summarize some perspectives to possibly break through them:

### 5.1 Li Source in Anode

There are three types of methods to preset the Li source into the anode, i.e., HC‐EM, DC‐EM, and CM. The as‐prepared pre‐lithiated anode with HC‐EM and DC‐EM are of high reactivity to the water even in the moisture free atmosphere and fresh electrolyte, which inevitably hinders their application. Thus, HC‐EM and DC‐EM are mainly restricted to lab studies. HC‐EM is an effective method to prepare pre‐lithiated anode to pair with cathode for the electrochemical evaluation of prototype full cell. DC‐EM can also play the same role as HC‐EM. Moreover, DC‐EM is also utilized to simulate the pre‐lithiation process as an extra Li source method. In comparison with those two strategies, CM has a greater potential for practical application. Also, the chemical stability of lithiated anode materials should be addressed.

### 5.2 Li Sources in Cathode

This method is a promising strategy to mitigate initial ALL on a practical level. However, the Li source should satisfy several requirements simultaneously, i.e., chemical stability, high capacity supplement, suitable potential range, and good conductivity and integrity. Also, if Li sources after releasing Li are in a gaseous state, the integrity of electrodes needs to be considered, i.e., the electrode microstructure needs to be carefully designed and tested when using such a method.

### 5.3 Sacrificed Electrode Method

This method has been widely utilized in LIC manufacturing but not in the field of LIBs. Different from the electrodes in LIC, the capacity of the battery electrode is several times higher and the electrodes are well matched so that pre‐lithiation treatment should be more uniform and accurate to ensure safe cycling. Therefore, it is necessary to control the pre‐lithiation degree and pre‐lithiation rate carefully, which involves the pore density and pore size design on the current collector and electrode, the rest time optimization for ion diffusion to equilibrium, and another detailed parameter optimization.

### 5.4 Extra Li Source

The small particle size and relatively low purity (<98%) of SLMP brings some difficulties for coating and assembly, as well as a negative effect on the electrochemical performance, e.g., severe self‐discharge behavior. In the research of LICs, the thin Li foils were developed as a substitute to achieve a better pre‐lithiation outcome. To make a comparison, four different types of Li‐source structures are introduced, namely SLMP, thick Li strips, thin Li foil, and thin Li foil with pinholes. In order to compare pre‐lithiation methods under the same situation, Li loaded in experimental cells has the same weight compared to anode mass, therefore, the anode surface is partially covered by thick (45 mm) Li strips or uniformly covered by thin (15 mm) Li foil. The pin holes on thin Li foil help electrolyte flow to the electrode and gas overflow from the electrode. It is concluded that pre‐lithiation with Li foil can manufacture cells more efficient and safer than using SLMP and the high surface area of Li source (such as SLMP) results in a high pre‐lithiation rate since Li‐ion comes from Li surface.^[^ ^132^ ^]^ It needs to be pointed out that a large amount of Li is loaded to fully lithiate anode. However, the Li foil used in LICs is 15 mm or thicker, which is still not thin enough for pre‐lithiation due to the great production difficulties in the industry. The commercialization of thinner Li foil (<10 um) will pave the path for the practical application of this method.

## Conflict of Interest

The authors declare no conflict of interest.
